# Lymphomas of the Vulva: A Review of the MITO Rare Cancer Group

**DOI:** 10.3390/cancers16112102

**Published:** 2024-05-31

**Authors:** Francescapaola Magazzino, Cynthia Aristei, Anna Passarelli, Antonio Pierini, Ugo De Giorgi, Ruby Martinello, Lavinia Domenici, Sandro Pignata, Giorgia Mangili, Gennaro Cormio

**Affiliations:** 1 Complex Operating Unit Obstetrics and Gynaecology, Ospedale Civile di San Donà di Piave-Venezia, AULSS4 Veneto Orientale, 30027 San Donà di Piave, Italy; 2 Radiation Oncology Section, Department of Medicine and Surgery, Perugia General Hospital Sant’Andrea delle Fratte, University of Perugia, 06156 Perugia, Italy; cynthia.aristei@unipg.it; 3 Department of Urology and Gynecology, Istituto Nazionale Tumori IRCCS “Fondazione G. Pascale”, 80144 Napoli, Italy; anna.passarelli@istitutotumori.na.it (A.P.); s.pignata@istitutotumori.na.it (S.P.); 4 Division of Hematolgy and Clinical Immunolgy, Department of Medicine and Surgery, University of Perugia, 06156 Perugia, Italy; antonio.pierini@unipg.it; 5 Department of Medical Oncology, IRCCS Istituto Romagnolo per lo Studio dei Tumori (IRST) “Dino Amadori”, 47014 Meldola, Italy; ugo.degiorgi@irst.emr.it; 6 Department of Medical Sciences, Institute of Obstetrics and Gynecology, University of Ferrara, 44121 Ferrara, Italy; mrtrby@unife.it; 7 2nd Division of Obstetrics and Gynaecology, Azienda Ospedaliera Universitaria Pisana, University of Pisa, 56126 Pisa, Italy; lavinia.domenici@gmail.com; 8 Department of Obstetrics and Gynaecology, San Raffaele Scientific Institute, 20132 Milano, Italy; mangili.giorgia@hsr.it; 9 Gynecologic Oncoly Unit, IRCCS Istituto Tumori “Giovanni Paolo II”, 70124 Bari, Italy; gennaro.cormio@uniba.it; 10 Department of Interdisciplinary Medicine, University of Bari, 70124 Bari, Italy

**Keywords:** vulvar lymphoma, cutaneous T-cell/B-cell lymphoma, classification, immunophenotype, molecular genetic profiling

## Abstract

**Simple Summary:**

Vulvar lymphomas are uncommon diseases and account for less than 0.5% of gynecological cancers and 1.5% of all NHLs. The various types of lymphomas have recently been identified as separate diseases and not as morphological variations of the same disease, according to the World Health Organization classification. They often represent the secondary localization of systemic disease, and diffuse large B-cell lymphoma is the most common type. We also considered cases of vulvar T-cell lymphomas. The data analysis highlighted the difficulty of diagnosis and the need to resort to biopsy–histological examination, as well as molecular methods. The correctness of the diagnosis directly affects appropriate therapeutic management, which requires an adequate surgical approach that can be integrated with chemotherapy and radiotherapy.

**Abstract:**

Since they are very rare tumors, lymphomas of the vulva are often not properly recognized. Patients with vulvar lymphoma are generally elderly and the classical manifestation of the disease is a vulvar mass. No significant age differences have been found between primary and secondary lymphoma. To make a correct diagnosis, it is therefore necessary to use not only histological examination but also the genetic and molecular profile in order to establish optimal therapeutic management. Literature analysis confirm the good prognosis of this disease.

## 1. Introduction

After having achieved an international consensus among pathologists, hematologists, radiologists, and clinical oncologists, the 2008 and 2017 editions of the “World Health Organization (WHO) Classification of Tumours of the Hematopoietic and Lymphoid Tissues guidelines for malignant lymphoma diagnosis” [1,2] defined multiple entities, according to the REAL classification [3], which incorporated morphology, immunophenotyping, genomics and clinical features. In 2022, the fifth edition of the World Health Organization Classification of Haematolymphoid Tumours defined a systemic evolution of the prior classifications [1,4].

Since they account for 5% of all malignancies, malignant lymphomas constitute a heterogeneous group of diseases arising from clonal lymphocyte proliferation. A total of 10% are Hodgkin lymphomas (HLs) of classical and non-classical sub-types, while the majority (90%) are identified as non-Hodgkin lymphomas (NHLs), which include B-cell, T-cell, and natural killer (NK) cell types [5].

Immunohistochemistry is the only analysis that distinguishes between B- and T-cell lineages.

Non-Hodgkin lymphomas (NHLs) account for 70–80% of all lymphomas, and their classic clinical characteristic manifestation is typically in lymph node stations or in other lymphoid tissues, like bone marrow and spleen. In 20–24% of cases, the localization of lymphoma disease may also involve extranodal sites, such as the nervous central system, gastrointestinal tract, salivary glands, lung, thyroid, and skin [6].

Lymphomas are defined as “primary cutaneous” if the primary diagnosis is in the skin, in the absence of evidence of extra-cutaneous disease.

Primary cutaneous lymphomas represent a heterogeneous group of T-cell and B-cell (CTCLs and CBCLs) that exhibit different clinical behaviors and prognoses from histologically similar systemic lymphomas that can affect the skin secondarily. For this reason, they need different types of treatment.

Classification systems for non-Hodgkin lymphomas, such as the European Organization for Research and Treatment of Cancer (EORTC) classification for primary cutaneous lymphomas and the World Health Organization (WHO) classification for tumors of hematopoietic and lymphoid tissues, include primary cutaneous lymphomas as separate entities [7,8].

In developed countries, CTCLs constitute approximately 75%–80% of all primary cutaneous lymphomas, and CBCLs 20%–25% [7]. Despite their peculiar clinical and histological features, cutaneous CTCLs and CBCLs are somewhat difficult to study due to their rarity. Having a completely different clinical behavior and prognosis than morphologically similar nodal lymphomas that may have secondary localization in the skin, they need a different type of treatment [9].

The objective of this review is to provide gynecological oncologists and hematologists with a detailed and precise update based on the latest WHO classification and on the most recent clinical, pathological, and biological characteristics of lymphoma of the vulva, a rare and poorly known tumor.

## 2. Materials and Methods

**Figure 1:**
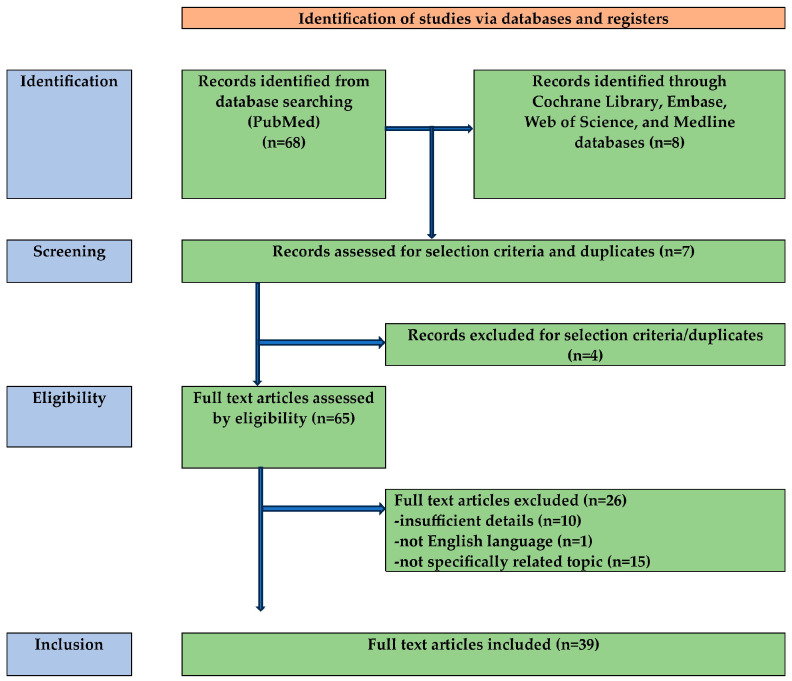
Study flow schema: PRISMA flow diagram of the process of identification, screening, and inclusion of articles. Systematic literature reviews were selected, using standard methods to be briefly presented in the article.

A systematic search of the literature until March 2024 was performed in the following electronic databases: PubMed, the Cochrane Library, Embase, Web of Science, and Medline databases. The article research was carried out according to the PRISMA (Preferred Reporting Items for Systematic Reviews and Meta-Analyses) framework [10], as presented in Figure 1. The protocol was not registered. In the article, it is possible to find the following search keywords, which were used alone or in combination: “Lymphomas”, “Vulvar lymphoma”, and “Cutaneous B-cell/T-cell Lymphomas”.

Information was derived from relatively large single-institution studies, multi-center consortium projects, and population-based registry analyses [11]. In particular, we considered case series and case reports published in English. For what concerns the publication period, no limitations were imposed. Two authors (F.M. and C.A.) independently reviewed the titles and abstracts of all of the eligible articles. Every possible duplicate was removed. The full texts of potentially suitable studies were then independently evaluated for eligibility by the two authors. Any discordance between the two authors was solved by discussion among the two senior reviewers (G.C. and G.M.).

Data were collected from the most relevant scientific articles published from 1937 to May 2021. Reviews or articles including lymphomas in the outside of the vulva and vulvar lymphomas in pregnant women were excluded. In total, 39 articles, for 54 cases, were selected to be included in this review and are reported in Table 1.

## 3. Incidence of Lymphomas of Female Genital Tract

Like all primary cutaneous lymphomas, the rare primary genital lymphoma may not be correctly diagnosed.

In women, genital lymphomas occur in different areas, such as the vulva, ovary, and uterine corpus. They are defined as primary if, at diagnosis, the following criteria are met:

(1) the disease is confined to the female genital tract, irrespective of the involvement of one or more sites (e.g., ovary and uterine corpus), with no evidence of disease elsewhere (full investigation failed to reveal any evidence of disease elsewhere);
(2) peripheral blood and bone marrow do not contain any abnormal cells indicating lymphoma;
(3) no remote organs are involved within 6 months of diagnosis.

Genital lymphomas are defined as secondary if they are diagnosed after or concurrent with lymph node detection [36].

Only 1.5% of NHLs are estimated to be primary lymphomas of the female genital tract, accounting for under 0.5% of gynecological cancers [6,50,51,52].

The prevalence order is ovary (49%), uterus (29%), fallopian tube (11%), vagina (7%), and vulva (4%) [6,25].

When there is a lack of evidence of extra-cutaneous disease, vulvar lymphomas are grouped as primary cutaneous lymphomas, which embrace distinct lymphoma entities [36]. They often display a completely different clinical profile and prognosis to histologically similar nodal lymphomas, which may involve the skin secondarily and require different treatment [4].

## 4. Clinical and Pathological Features of Vulvar Lymphoma

In spite of the common symptoms, lymphomas are different diseases in that they show clinical–pathological features and prognoses [53], as reported in the literature and described in Table 1.

Patients affected by vulvar lymphoma are older than patients with lymphomas that affect any other gynecological site. No significant age differences have been found between primary and secondary lymphoma.

On a clinical level, vulvar lymphomas occur with swelling, itching, burning, discomfort, or pain, usually associated with fast-growing, nontender, and ulcerated lesions. Edema may concern only the lesion site or the entire vulva, depending on the size of the lesion.

If not recognized and consequently not adequately treated, it can extend so much that it subverts and destroys the anatomy of the vulva and perineum.

## 5. Results

In our review, fifty-four cases of vulvar lymphoma were reported in Table 1, with a median age of 50 years (range: 13–89). The most common anatomical site of interest was the vulva, although there are cases also involving the perineum, buttocks, and vagina. The mean lesion size at the time of treatment was 6 cm (data available for 23 cases, range 1.0–20.0 cm). Vulvar lymphoma has usually been described as a mass, with or without ulceration, and it has rarely been associated with pain, in only one case with fever, more frequently with discomfort in a growing mass.

Different immunophenotypes were reported. As in other genital sites, B-cell NHL is the most frequent (28/54 = 51.8%), with DLBCL the predominant histologic type (16/54 = 29.6%). The T-cell immunophenotype occurs less frequently (10/54 = 18.5%), but in some cases (16/54 = 29.6%), the immunophenotype is unknown.

Literature analysis shows that primary tumors are more numerous than secondary vulvar localizations (primary: 40/54 = 74%, secondary: 14/54 = 26%), and the most frequent treatment is chemotherapy with (16/54 = 29.6%) or without (11/54 = 20.4%) other therapies.

Literature analysis and data in Table 1 confirm the good prognosis of this disease. In fact, the patients who survived, in variable periods of observation, were 27/54 (50%), and only 8/54 (14.8%) died of disease, while 2/54 (3.7%) died of other causes and 3/54 (5.5%) of unknown causes. We have no follow-up data for 14/54 patients (26%).

## 6. Data Discussion

Patients with vulvar lymphoma are generally elderly (the mean age is about 60 years, range: 43–71 years) [33], and the classical manifestation of the disease is a vulvar mass.

On a histological level, B-cell lymphomas are the predominant type, whereas only a few cases of T-cell lymphoma have been described. The most common type is mycosis fungoides, although it occurs as a secondary involvement of the vulva in patients affected by NHL [33].

Although vulvar lymphomas usually affect the major or minor labia, two cases have been found in the Bartholin’s gland: one secondary, described by Plouffe, with Bartholin’s gland involvement by primary diffuse histiocytic lymphoma, found in the right breast, and one primary, described by Tjalma, considered the first case in the literature of primary diffuse large B-cell non-Hodgkin lymphoma involving the Bartholin’s gland) [21,35]. Ferrando-Marco reported one primary malignant large cleaved cell vulvar lymphoma, predominantly involving the clitoris, in a 60-year-old woman [27].

Martorell [37] described the case of an elderly woman with a lymphoma-like lesion of the left major labia, which, 7 years after diagnosis, transformed into a marginal zone B-cell lymphoma (lymphoplasmacytic lymphoma). In contrast with the other two studies in the literature, no correlation emerged with Epstein–Barr virus/Borrelia burgdorferi infection, as PCR detection of Borrelia burgdorferi and Epstein–Barr virus DNA was negative. This emerged even though marginal zone B-cell lymphoma has usually been associated with the use of drugs, such as fluoxetine and amitriptyline, which can alter the activity of the lymphoid system. El Kacemi [43] described the only case of primary NHL in a young immunocompetent woman. All the other cases in the literature involved patients who were immuno-deficient (e.g., with iatrogenic immunosuppression after allograft or with HIV infection) [30,31,54].

Even though Winnicki [38] described a case of vulvar T-cell HL of the vulva in a patient with Crohn’s disease, a significant increase in the incidence of vulvar lymphoma has not been detected in patients with inflammatory bowel disease. Despite this case report, Hodgkin lymphoma in patients with Crohn’s disease remains rare and most commonly affects the intestine [55,56,57]. So far, no clear link between Crohn’s disease and Hodgkin lymphoma has been found in any study; conversely, Palli, in his population-based study, demonstrated a correlation with an increased risk of ulcerative colitis [58].

Buras [42] described, in a 50-year-old woman, the first case of primary mycosis fungoides of the vulva. Flow cytometry analysis revealed a predominant T-cell (CD2+, CD3+, CD5+) population that expressed CD4 and was negative for CD8. Immunohistochemical analysis revealed a T-cell population that was negative for natural killer cell markers. There was a minor lymphoid component in the lesion that was positive for CD20, a B-cell marker.

Recently, Dashraath [48] observed a rare cutaneous γ/δ T-cell subtype lymphoma in an elderly patient with diabetes.

Zizi-Sermpetzoglou [40] described the case of a 48-year-old woman who presented with primary intravascular vulvar lymphoma of T-cell origin and CD30 positive, while Morse [49] recently disclosed a case of a 64-year-old woman with aggressive primitive epidermotropic cytotoxic T-cell lymphoma, successfully treated with RT.

## 7. Immunophenotypic and Molecular Profiling

Histopathological classification and molecular profiling are essential for the diagnosis and treatment of lymphomas.

The use of immunohistochemistry and molecular genetic analyses makes it possible to distinguish between lymphoma, rhabdomyosarcoma, Ewing’s sarcoma/peripheral neuroectodermal tumor (PNET), and Merkel cell tumor, an aggressive neoplasm of skin, which may rarely occur on the vulva [45].

In addition, reactive vulvar processes usually manifest as surface-extension, band-like, and non-infiltrating and commonly have a polymorphous population of lymphocytes, plasma cells, and histiocytes without atypia.

The hematoxylin–eosin-stained histologic sections of the vulvar masses’ biopsy showed diffuse lymphomatous infiltrate cells with round nuclear contours, dispersed chromatin, small nucleoli, and scant cytoplasm. There were numerous mitotic figures and apoptotic bodies, and there were scattered “tingible body” macrophages containing nuclear and other cell debris dispersed amongst the tumor cells, leading to a starry-sky appearance [45,54]. The presence or absence of fibrosis, necrosis, granulomas, and variations in cell morphology should also be noted.

From a morphologic standpoint, DLBCL is defined as a diffuse growth of neoplastic large B lymphoid cells that have a nuclear size equal to or exceeding normal macrophage nuclei, with prominent central nucleoli or smaller nucleoli, or obviously anaplastic.

Gene expression profiling has identified two major distinct forms of DLBCL depending on the cell-of-origin (COO): the germinal center B-cell (GCBC-) and the activated B-cell (ABC-) types. The former appears to derive from germinal center B cells, while the latter may derive from a post-germinal center B-cell (COO). ABC-DLBCL, which was associated with increased nuclear factor kB activity in the kB NF-kB chain, is characterized by more aggressive behavior and a worse prognosis than GBC DBCL [59,60,61,62].

Consequently, distinguishing between ABC and GBC DLBCLs is of clinical importance because, while the majority of patients with GCB-DLBCL respond well to standard R-CHOP (rituximab, cyclophosphamide, doxorubicin, vincristine, and prednisone) regimen chemotherapy, patients with ABC-DLBCL have a poorer prognosis.

The B-cell lineage is CD20-positive and CD30-negative, while vice versa, the T-cell lineage is CD30-positive and CD20-negative. Additionally, a screening immunohistochemical panel of other markers, such as PAX-5, CD79, MUM1, is available for B-cell lymphomas [33,36,42,47,54,63,64].

More recently, studies on the role of key gene mutations and gene pathways helped to elucidate molecular mechanisms of lymphoma development and provided new prognostic tools to the clinician [65].

One clear example is the role of MYC alterations. Indeed, MYC rearrangements were found in 5% to 15% of DLBCLs and NOS often showed up with BCL2 or, less frequently, with BCL6 translocation [66,67]. Transcription factor TCF3 or its negative regulator ID3 was associated with mutations in approximately 70% of sporadic and immunodeficient-related Burkitt lymphoma [68,69,70,71].

As for T-cell lymphomas, genetic studies have shown the correlation with reference antigens, specifically CD279/PD1, CD10, BCL6, CXCL13, ICOS, SAP, and CCR5, but also genetic abnormalities included TET2, IDH2, DNMT3A, RHOA, and CD28 mutations, or gene fusions (for example ITK-SYK or CTLA4-CD28) [65].

More recent studies highlighted JAK/STAT pathway mutations in several T-cell and NK-cell tumors. The importance of these anomalies lies in their correlation with the lymphomagenesis process, with the precise identification of the disease and its clinical severity, and with the possibility of setting up targeted therapies [65,72,73,74,75].

## 8. Instrumental Investigations

The diagnosis of lymphomas, including the vulvar, makes use of the molecular identification of cellular strains that determine the disease. Biopsy findings indicate the type of vulvar lymphoma and appropriate approaches, from instrumental investigations to treatment.

Imaging studies play a major role in diagnosis, disease staging, and follow-up. Chest, abdomen, and pelvis CT scan or MRI are required for vulvar lymphoma staging, according to the Ann Arbor classification system and the IPI (International Prognostic Index) [76,77]. Bone marrow biopsy may also be useful in staging and in differentiating primary from secondary forms. In conjunction with it, positron emission tomography (PET) imaging aids in correctly staging the disease. In fact, PET/CT is more accurate and sensitive than CT scans in identifying lymph nodes and extranodal localizations. PET-CT and MRI indicate more precisely whether surgery is appropriate. Basal CT, PET-CT, and MRI imaging play a key role not only in defining the disease stage at diagnosis and in providing baseline measurements for comparison purposes but also in setting and evaluating the treatment response.

It is interesting to note that the end-of-treatment PET complete response is highly predictive of progression-free survival and overall survival in DLBCL after first line immunochemotherapy. The suspicion of lymphoma is particularly strong when one or more solid, well-defined, homogeneous masses are displayed without necrosis, regardless of size or diffuse infiltration with architectural preservation. In addition, pelvic lymphadenopathy may be evident [78]. Lymphoma density on CT scans is similar to moderately hypo-attenuated muscles when compared with the surrounding parenchyma but more attenuated than water.

On MRI, lymphomas usually show low-to-intermediate signal intensity on T1-weighted imaging and moderately high signal intensity on T2-weighted imaging.

On PET, a standardized uptake value greater than 2.5 suggests malignancy [78]. PET imaging, in combination with bone marrow biopsy, contributes to the correct staging of disease. Basal imaging serves both to define the disease stage at diagnosis and to provide baseline measurements for comparison to evaluate treatment response.

## 9. Treatment

If the rarity of primary and secondary vulvar lymphomas is considered, there is currently no consensus on the treatment. Consequently, patients are initially treated for any other common gynecologic malignancy, and treatment is fundamentally based on the surgical and radio/chemotherapy approach. In clinical practice, the treatment decision is also determined based on various factors such as the stage, age, presence of bulky disease, performance status, and International Prognostic Index (IPI). IPI is the most commonly used model for predicting survival in aggressive lymphoma.

Vulvar NHL has a good prognosis, and a complete cure has been demonstrated. Indeed, NHLs are generally highly responsive to radio-chemotherapy; thus, surgery is not the primary choice of therapy for vulvar lymphoma.

For early stage I and II disease, radiotherapy alone is suitable, providing good overall results. The dose range varies from 30 to 40 Gy in 3–4 weeks [43,79]. For advanced stage III and IV disease, the most frequent approach consists of 4–6 cycles of R-CHOP (rituximab, cyclophosphamide, doxorubicin, vincristine, and prednisone) chemotherapy, with or without radiotherapy consolidation. This treatment is particularly indicated, as it achieved a 60–70% complete response rate [6]. Therefore, chemotherapy produces approximately the majority remission rate, but relapse of the disease, even after complete remission, is not unusual in these highly malignant forms, especially in the central nervous system. When these relapses occur, the outcome will be fatal.

R-CHOP appears more effective in patients with GCB-DLBCL than with ABC-DLBCL. In the latter setting, radiotherapy may improve response.

Finally, radiotherapy can be administered with a symptomatic, palliative aim or in the management of aggressive cutaneous vulvar lymphomas, as the vulvar primary cutaneous CD8+ aggressive epidermotropic cytotoxic T-cell lymphoma was reported to be highly responsive to radiation therapy [49].

## 10. Conclusions

Although vulvar lymphoma has been reported to involve the female genital tract, vulvar localization remains extremely rare. Hodgkin lymphomas (HLs), with their classical and non-classical sub-types, represent the minor component, while the majority are identified as non-Hodgkin lymphomas (NHLs), which include B-cell, T-cell, and natural killer (NK) cell types.

Primary vulvar lymphomas usually present as a fast-growing, nontender, and ulcerated vulvar mass, often associated with nonspecific symptoms such as itching, swelling, itching, burning, discomfort, or pain, usually associated with edema that may involve only the lesion site or the entire vulva. They are aggressive and, from a pathological point of view, are most often DLBCLs.

Diagnosis requires biopsy, and management may involve a combination of surgery, radiotherapy, and chemotherapy. Since vulvar lymphomas are rare tumors, management by a multidisciplinary team is highly recommended, and experts in hematological diseases should be involved in disease management.

## Figures and Tables

**Table 1:** Lymphomas of the vulva: main clinical and pathological features reported in the literature.

Reference	Sample Size, *n*	Histological Type	Immuno- phenotype	Primary (P)/ Secondary (S)	Age	Presentation (Size)/Site	History of NHL/Other	Ann Arbor Stage	Therapy	Response/ Follow-Up (Months)
Taussig FJ, 1937 [12]	1	NA	NA	P	63	Vulvar mass (2 × 1.5 cm)	None	NA	Local excision	Death at 1 month (other cause)
Buckingham JC, 1955 [13]	1	Reticulum cell sarcoma	NA	P	33	Vulvar mass (14 cm)	None	NA	Local excision—RT	Alive at 6 months; RD
Borglin NE, 1966 [14]	1	Histiocytosis X	NA	P	42	Area of infiltration (6 × 3 × 1.5 cm)	None	NA	Vulvectomy	NA
Iliya FA, 1968 [15]	1	Reticulum cell sarcoma	NA	P	75	Vulvar mass (3 cm)	None	NA	Local excision	Alive-NED at 60 months
Schiller HM, 1970 [16]	1	DLCL	NA	S	89	Vulvar lesion (5 × 4 cm)	NA	IV	Not performed	Death at 5 days (other cause)
Sneddon I, 1972 [17]	1	Reticulum cell sarcoma	NA	P	42	Vulvar ulceration	None	NA	RT	NA
Doss LL, 1978 [18]	1	Plasmocytoma	NA	P	NA	NA	NA	NA	NA	CR/Alive at 60 months
Igarashi M, 1979 [19]	1	Follicular small, cleaved cell	NA	S	72	Cutaneous nodules of vulva, anal region, and anxillae	NA	IV	RT, steroids	CR/Death at 3 months (cause NA)
Egwuatu VE, 1980 [20]	1	Burkitt lymphoma	NA	P	13	Vulvar mass, ulceration	None	NA	Chemotherapy	CR
Plouffe L, 1984 [21]	1	DLCL	NA	S	66	Enlarged bilateral Bartholin’s glands	DLCL	IV	Chemotherapy	PD/Death at 1 month (cause NA)
Swanson S, 1987 [22]	1	PTCL	T-cell	P	76	Vulvar nodule (6 cm)	None	IE	Chemo-RT, surgery	PD/DOD at 3 months
Tome MA, 1987 [23]	1	Diffuse, mixed	NA	P	45	Vulvar mass (6 cm)	None	IIE	Chemo-RT	CR/NED at 18 months
Mikhail MS, 1989 [24]	1	Small lymphocytic	NA	S	74	Ulcerated lesion	CLL	IV	Chemotherapy	PR/Alive at 24 months
Bagella MP, 1990 [25]	1	DLCL	NA	P	61	Vulvar mass	None	IE	Chemo-surgery	CR/NED at 10 months
Tuder RM, 1992 [26]	1	LPL	NA	P	21	Vulvar mass	None	NA	Chemo-surgery	DOD at 3.3 months
	1	Angiocentric small and large mixed-cell lymphoma	NA	P	49	Perineal induration	None	NA	Chemotherapy	PD with response to Chemo; alive at 18 months
Ferrando-Marco J, 1992 [27]	1	FLCL	B-cell	P	60	Vulvar mass (clitoris + bilateral labia minora)	None	IE	Chemotherapy	NED at 36 months
Marcos C, 1992 [28]	1	DLBCL	B-cell	P	79	Vulvar mass (6 cm)	None	IE	RT	CR/NED at 10 months
Nam JH, 1992 [29]	1	DLBCL	B-cell	P	68	Vulvar mass (6 cm)	None	IE	Surgery	NED at 14 months
Kaplan MA, 1993 [30]	1	T-cell lymphoma	T-cell	P	55	Vulvar mass (5 cm)	None	IE	Not performed	Death at 11 days (cause NA)
Kaplan EJ, 1996 [31]	1	DLBCL	B-cell	P	25	Vulvar mass (10 × 10 × 12 cm)	None	IE	Chemo-RT	PR; DOD at 7 months
Amichetti M, 1999 [32]	1	DLCL	NA	S	74	NA	DLCL	IV	Chemo-surgery	DOD at 6 months
Vang R, 2000 [33]	2	DLBCL	B-cell	P	67	Vulvar mass	None	IIE IE	Chemo-RT NA	RD in spine; DOD at 24 months NA
	1	MF	T-cell	S	43	No mass	MF	IV	Chemo-phototherapy	Initial CR; after, RD and alive with disease at 48 months
	1	DLBCL	B-cell	S	56	Vulvar mass (1 cm)	DLBCL	IV	Chemo-RT	Initial PR; after PD with central nervous system /bone involvement/DOD at 7 months
	1	PTCL	T-cell	S	56	Right labial nodules	Large T-cell NHL	IV	Chemotherapy	PD/DOD at 60 months
	1	DLBCL	B-cell	P	68	Vulvar-vaginal mass with ulcer (>7 cm)	None	NA	NA	NA
Iczkowski KA, 2000 [34]	1	NA	B-cell	P	64	Induration and ulceration (3 × 2 cm)	None	NA	Chemotherapy	CR/Alive at 12 months
Tjalma WA, 2002 [35]	1	DLBCL	B-cell	P	73	Vulvar mass (3 × 1.5 cm)	None	IE	Local excision-RT; Chemotherapy	RD at 6 months; CR-NED at 51 months
Kosari F, 2005 [36]	3	DLBCL	B-cell	1P-2S	>19	NA	NA	Localized	NA	NA
	1	FL grade I	B-cell	1S	>19	NA	NA	NA	NA	NA
	4	LPL	B-cell	2P-2S	>19	NA	NA	NA	NA	NA
Signorelli M, 2006 [6]	1	FL grade 3	B-cell	P	75	Vulvar mass (3 × 2 cm)	NA	IIE	Chemotherapy	CR/NED at 247 months
	1	DLBCL	B-cell	P	75	Vulva	NA	IE	Chemotherapy	CR/NED at 21 months
Martorell M, 2008 [37]	1	LPL	B-cell	P	80	Vulvar nodule (1 cm)	Vulvar pseudo-lymphoma	NA	Asiaticoside + RT	CR/NED at 36 months
Winnicki M, 2009 [38]	1	HL	T-cell	S	45	Perineum mass involving majora, minora labia, and the clitoris (20 × 20 cm)	Crohn disease	IV	Chemotherapy—RT	PR
Koh LP, 2009 [39]	1	c-anaplastic large cell lymphoma	T-cell	P	35	Ulcerated vulvar mass (8 × 5 cm)	None	NA	Chemotherapy—RT	CR/Alive at 15 months
Zizi-Sermpetzoglou A, 2009 [40]	1	T-cell	T-cell CD30^+^	P	48	Fever and a slow-growing mass of the vulva	None	Intravascular vulvar lymphoma	Local excision	CR
Plaza JA, 2011 [41]	1	PLBCL	B-cell	P	NA	NA	None	IE	NA	NA
Buras AL, 2015 [42]	1	MF	T-cell	P	50	Redundant labia	NA	NA	Surgery	CR/NED at 156months
El Kacemi H, 2015 [43]	1	DLBCL	B-cell	P	37	Ulcerated vulvar mass (13 × 7 cm)	None	IIE	Chemotherapy—RT	CR/NED at 36 months
Clemente N, 2016 [44]	1	DLBCL	B-cell	P	43	Vulvar mass (3 cm)	Vulvar pseudo-lymphoma	IIE	Local excision—Chemotherapy	CR/NED at 6 months
Wang Q, 2017 [45]	1	DLBCL	B-cell	P	70	Vulvar polypoid mass (3 cm)	None	IV	Chemotherapy	NA
Kanis MJ, 2018 [46]	1	MZL	B-cell	P	61	Vulvar mass (5 × 3 cm)	None	NA	Chemotherapy	PR/SD
Ye AL, 2018 [47]	1	PCDLBCL- LT;	B-cell	P	38	Ulcerated vulvar mass	None	IE	RT	CR/NED at 84 months
	1	DLBCL	B-cell	P	73	Ulcerated vulvar mass	None	IE	Local excision	CR/NED at 65 months
Dashraath P, 2020 [48]	1	γ/δ T-cell lymphoma	T-cell	P	86	Vulvar mass ulceration	None	NA	Palliative RT	PD/ DOD
Morse DC, 2021 [49]	1	AECTCL	T-cell CD8+	P	64	Left buttock nodule-perineal skin	None	NA	Oral bexarotene+ psoralene+ ultraviolet; RT	PD Initial PR; after, PD with left breast lesion: CR/ NED at 6months

**Table legend:** NA, not available; RT, radiotherapy; DLCL, diffuse large cell lymphoma; HL, Hodgkin lymphoma; NHL, Non-Hodgkin lymphoma; LPL, lymphoplasmacytic lymphoma; DLBCL, diffuse large B-cell lymphoma; CLL, chronic lymphocytic leukemia; FL, follicular lymphoma; FLCL, follicular large cell lymphoma; MF, mycosis fungoides; PTCL, peripheral T-cell lymphoma. MZL, marginal zone lymphoma; PLBCL, primary cutaneous large B-cell lymphomas; PCDLBCL-LT, primary cutaneous diffuse large B-cell lymphomas-leg type; AECTCL, aggressive epidermotropic cytotoxic T-cell lymphoma; PD, progression disease; RD, relapsed disease; PR, partial response; SD, stable disease; CR, complete response; DOD, died of disease; NED, no evidence of disease.
